# A multidomain lifestyle intervention to maintain optimal cognitive functioning in Dutch older adults—study design and baseline characteristics of the FINGER-NL randomized controlled trial

**DOI:** 10.1186/s13195-024-01495-8

**Published:** 2024-06-13

**Authors:** Kay Deckers, Marissa D. Zwan, Lion M. Soons, Lisa Waterink, Sonja Beers, Sofie van Houdt, Berrit Stiensma, Judy Z. Kwant, Sophie C. P. M. Wimmers, Rachel A. M. Heutz, Jurgen A. H. R. Claassen, Joukje M. Oosterman, Rianne A. A. de Heus, Ondine van de Rest, Yannick Vermeiren, Richard C. Oude Voshaar, Nynke Smidt, Laus M. Broersen, Sietske A. M. Sikkes, Esther Aarts, MOCIA consortium, FINGER-NL consortium, Sebastian Köhler, Wiesje M. van der Flier

**Affiliations:** 1 Department of Psychiatry and Neuropsychology, Alzheimer Centrum Limburg, Mental Health and Neuroscience Research Institute (MHeNs), Maastricht University, PO Box 616, 6200 MD Maastricht, The Netherlands; 2 Alzheimer Center Amsterdam, Neurology, Vrije Universiteit Amsterdam, Amsterdam UMC Location VUmc, Amsterdam, The Netherlands; 3 Amsterdam Neuroscience, Neurodegeneration, Amsterdam, The Netherlands; 4 Division of Human Nutrition and Health, Wageningen University & Research, Wageningen, The Netherlands; 5 HAN University of Applied Sciences, Nijmegen, The Netherlands; 6 Department of Epidemiology, University of Groningen, University Medical Center Groningen, Groningen, The Netherlands; 7 Department of Geriatrics, Radboud University Medical Center, Radboudumc Alzheimer Center, Nijmegen, The Netherlands; 8 Radboud University, Donders Institute for Brain, Cognition and Behaviour, Nijmegen, The Netherlands; 9 Department of Cardiovascular Sciences, Leicester University, Leicester, UK; 10 Department of Primary and Community Care, Radboud University Medical Center, Nijmegen, The Netherlands; 11 Department of Psychiatry, University of Groningen, University Medical Center Groningen, Groningen, The Netherlands; 12 Danone Research & Innovation, Utrecht, The Netherlands; 13 Department of Clinical, Neuro and Developmental Psychology, VU University, Amsterdam, The Netherlands; 14 Epidemiology & Data Science, Vrije Universiteit Amsterdam, Amsterdam UMC Location VUmc, Amsterdam, The Netherlands

**Keywords:** Prevention, Cognitive impairment, Dementia, Alzheimer’s disease, Multidomain, Lifestyle, Risk factors, Intervention, Randomized controlled trial

## Abstract

**Background:**

Evidence on the effectiveness of multidomain lifestyle interventions to prevent cognitive decline in older people without dementia is mixed. Embedded in the World-Wide FINGERS initiative, FINGER-NL aims to investigate the effectiveness of a 2-year multidomain lifestyle intervention on cognitive functioning in older Dutch at risk individuals.

**Methods:**

Multi-center, randomized, controlled, multidomain lifestyle intervention trial with a duration of 24 months. 1210 adults between 60–79 years old with presence of ≥ 2 modifiable risk factors and ≥ 1 non-modifiable risk factor for cognitive decline were recruited between January 2022 and May 2023 via the Dutch Brain Research Registry and across five study sites in the Netherlands. Participants were randomized to either a high-intensity or a low-intensity intervention group. The multidomain intervention comprises a combination of 7 lifestyle components (physical activity, cognitive training, cardiovascular risk factor management, nutritional counseling, sleep counseling, stress management, and social activities) and 1 nutritional product (Souvenaid®) that could help maintain cognitive functioning. The high-intensity intervention group receives a personalized, supervised and hybrid intervention consisting of group meetings (on-site and online) and individual sessions guided by a trained lifestyle coach, and access to a digital intervention platform that provides custom-made training materials and selected lifestyle apps. The low-intensity intervention group receives bi-monthly online lifestyle-related health advice via the digital intervention platform. Primary outcome is 2-year change on a cognitive composite score covering processing speed, executive function, and memory.

**Results:**

Within 17 months, participant recruitment has been successfully completed (*N* = 1210; mean age: 67.7 years (SD: 4.6); 64% female). Modifiable risk factors commonly present at baseline were physical inactivity (89%), low mental/cognitive activity (50%), low social engagement (39%), hypertension (39%) and high alcohol consumption (39%). The mean body mass index of participants was 28.3 (SD: 4.2) and the total serum cholesterol was 5.4 mmol/L (SD: 1.2).

**Conclusions:**

Baseline lifestyle and clinical measurements showed successful recruitment of participants with sufficient potential for prevention. Results of FINGER-NL will provide further insight into the efficacy of a multidomain lifestyle intervention to prevent cognitive decline in older adults.

**Trial registration:**

ClinicalTrials.gov (ID: NCT05256199)/2022–01-11.

**Supplementary Information:**

The online version contains supplementary material available at 10.1186/s13195-024-01495-8.

## Background

Worldwide, more than 55 million people have dementia and this number is expected to increase with nearly 10 million each year [1]. About 40% of dementia cases have been estimated to be attributable to twelve modifiable factors (including e.g. midlife hypertension, midlife obesity, physical inactivity), which provides opportunities for prevention [2, 3]. In the last decade, research has identified additional modifiable factors such as poor sleep quality, low social contact, and psychological stress [4]. Single-domain intervention studies targeting lifestyle factors to prevent cognitive decline and dementia have yielded mainly non-significant results, although some small positive effects on cognition have been reported for dietary intervention, physical activity and cognitive training [5].

Studies on successful prevention of cardiovascular disease and type-2 diabetes have emphasized the importance of a multidomain lifestyle approach [6, 7]. The Finnish Geriatric Intervention Study to Prevent Cognitive Impairment and Disability (FINGER) study was the first large, long-term randomized controlled trial demonstrating that simultaneously targeting four lifestyle domains (physical activity, cognitive training, nutritional counselling and cardiovascular risk management) had a small but significant effect on cognition in older adults at-risk for dementia [8]. In contrast, the Multidomain Alzheimer Preventive Trial (MAPT; 3-year change in memory function) and the Dutch Prevention of Dementia by Intensive Vascular Care (preDIVA; incident dementia) did not reach their primary endpoint, but showed potential beneficial effects on cognition in specific subgroups of older people with increased risk of dementia [9, 10]. The more recent German AgeWell trial did not detect overall beneficial effects of a multidomain intervention on global cognitive performance, yet showed some promising effects in subgroups [11]. Notably, the COVID-19 pandemic imposed serious challenges on the execution of this trial. Therefore, it has become clear that new-generation interventions have to consider the possible impact of pandemics on recruitment, participation and adherence, for example, by using digital tools and online or hybrid intervention sessions.

Inspired by the results of FINGER, the World-Wide FINGERS network was established to adapt the original FINGER trial design to local circumstances [12]. In anticipation of future tailored intervention approaches, FINGER-NL combines multidomain lifestyle modifications and a nutritional intervention. Souvenaid® is a nutritional product designed to address specific nutritional needs in individuals in the early stages of Alzheimer’s disease and has been shown to have neuroprotective properties [13, 14]. In addition, based on promising findings in previous studies, we added two more lifestyle domains to the hybrid intervention, i.e., sleep counseling [15, 16] and stress management [17, 18], and intensified the social activities domain [19], resulting in a broader and more holistic intervention.

The primary objective of FINGER-NL is to investigate the effectiveness of a personalized, 2-year multidomain lifestyle intervention (high-intensity intervention group; HI-group) compared to online lifestyle-related health education (low-intensity intervention group; LI-group) on change in cognition in older adults at risk of cognitive decline. Secondary outcomes include 2-year change on individual cognitive tests, instrumental activities of daily living, quality of life, a modifiable dementia risk score (LIBRA) previously shown to be sensitive to the MAPT, preDIVA and FINGER multidomain interventions [20, 21], intervention-specific outcomes and blood-based biomarkers for Alzheimer’s disease. We also investigate heterogeneity in treatment effects on primary and secondary outcome measurements by baseline characteristics.

## Methods

### Study design

**Fig. 1:**
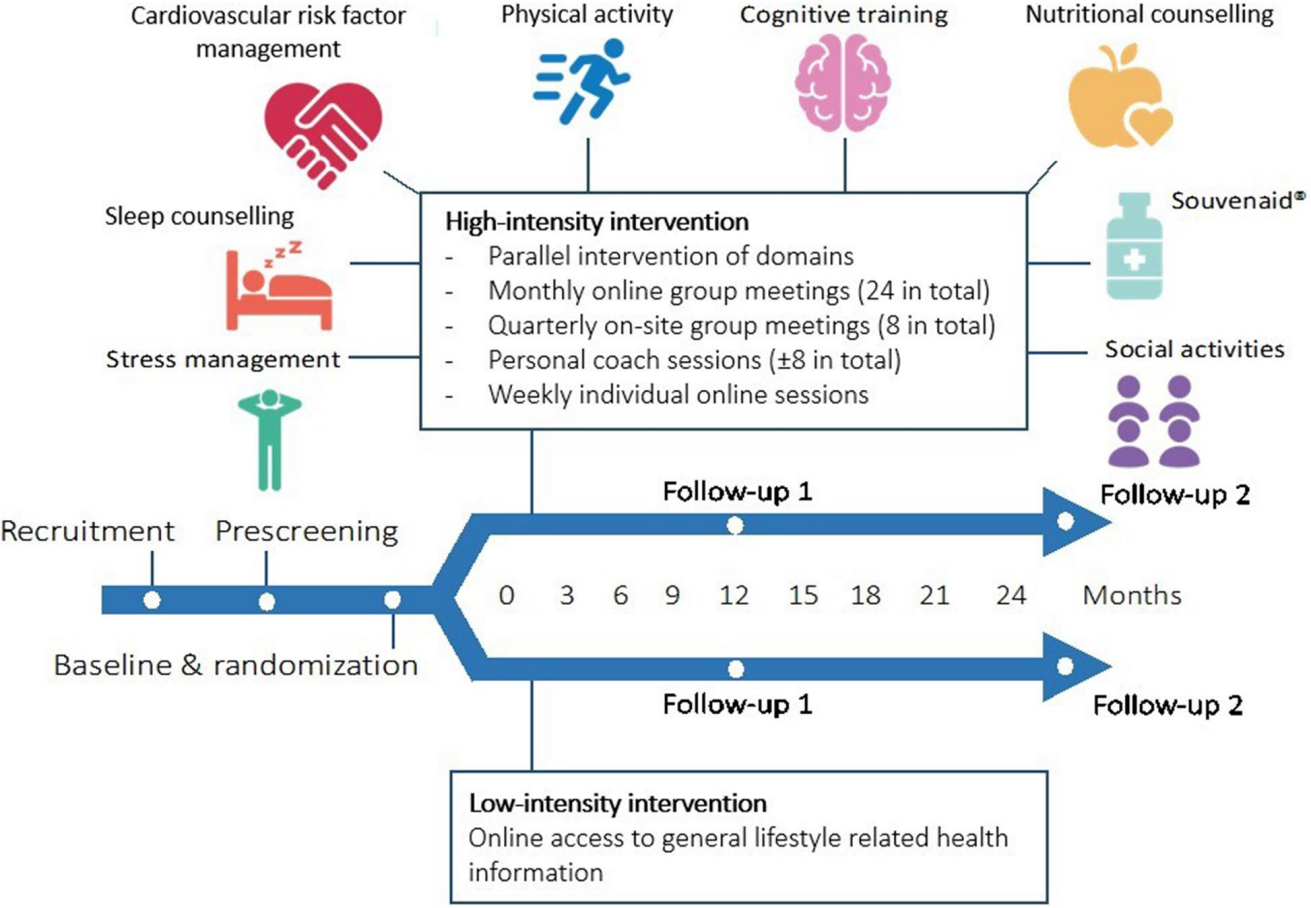
Summary of the FINGER-NL study design

FINGER-NL is a multi-center, randomized, controlled, multidomain lifestyle intervention trial among 1,210 older adults at risk for cognitive decline, with a duration of 24 months. Block-randomization stratified by study site (block sizes of preferably 20–24 participants) was used to centrally allocate eligible participants in a 1:1 ratio to a personalized multidomain lifestyle intervention (HI-group) versus online access to general lifestyle-related health information (LI-group). The multidomain intervention comprises a combination of 7 lifestyle components (physical activity, cognitive training, cardiovascular risk factor management, nutritional counseling, sleep counseling, stress management, social activities) and a nutritional product (Souvenaid, 125 mL). The study is conducted in five study centers across The Netherlands (Amsterdam, Groningen, Maastricht, Nijmegen, Wageningen). Outcome measurements are conducted at baseline, 12 months (Follow-up 1) and 24 months (Follow-up 2) after randomization. A flow-chart of the study design is shown in Fig. 1. FINGER-NL is part of the ‘Maintaining Optimal Cognitive Function In Ageing’ (MOCIA) research program (https://mocia.nl/scientific/).

### Recruitment of participants

Participant recruitment took place via the online recruitment platform Dutch Brain Research Registry (https://hersenonderzoek.nl/) which supports recruitment and pre-screening of research volunteers for neuroscience studies through a fully online registry [22]. In parallel, study sites used their own local participant pools and deployed initiatives for additional recruitment.

### Inclusion criteria

Inclusion criteria were (1) age 60–79 years at pre-screening; (2) adequate fluency in Dutch to understand the informed consent and complete study questionnaires; (3) informed consent to all study procedures; (4) Internet access at home; (5) presence of ≥ 3 self-reported risk factors for cognitive decline (including at least 2 modifiable risk factors and 1 non-modifiable risk factor). Modifiable risk factors include self-reported presence (based on a single question) of risk factors [23, 24].

**Table:** 

**Modifiable risk factors:** - Physical activity - Unhealthy diet - Low mental/cognitive activity - High blood pressure - High cholesterol - High body mass index (defined as ≥ 25 kg/m^2^ for 60–69 years old, and ≥ 28 kg/m^2^ for ≥ 70 years old, based on self-reported height and weight) **Non-modifiable risk factors:** - First-degree family history of dementia - Subjective cognitive decline/memory complaints	

### Exclusion criteria

Exclusion criteria were: (1) self-reported diagnosis of dementia or mild cognitive impairment; (2) cognitive impairment assessed by the Modified Telephone Interview for Cognitive Status battery (TICSm score < 23) [25]; (3) conditions affecting safe and continuous engagement in the intervention (e.g. under treatment for current malignant diseases), major psychiatric disorders (e.g. major depression, psychosis, bipolar disorder), neurological disorders thought to interfere with cognitive function (e.g. Parkinson’s disease, multiple sclerosis), symptomatic cardiovascular disease (e.g. stroke, angina pectoris, heart failure, myocardial infarction), re-vascularization within the last three months, severe loss of vision, impaired hearing or communicative ability, severe mobility impairment, other conditions preventing co-operation as judged by the local study nurse or consulted physician at the local study site; (4) simultaneous participation in any other intervention trial at time of pre-screening; (5) participation in FINGER-NL of another household member to prevent contamination.

### Online (automatic) prescreening and telephone prescreening

Online (automatic) prescreening was performed in potentially eligible participants recruited through the Dutch Brain Research Registry according to inclusion and exclusion criteria (except for TICSm score) to reduce screen failures. For individuals recruited through local cohorts, manual prescreening was performed based on available information on inclusion and exclusion criteria. On first contact, participants received the screening information letter, study information letter and informed consent form. In a second step, interested participants underwent an eligibility-check and a brief cognitive assessment (TICSm) via telephone screening performed by the five FINGER-NL study sites [25]. If participants fulfilled all study criteria, they were invited for an on-site baseline visit.

### Randomization and blinding

Participants were randomized after providing written informed consent and after completing the baseline visit. Participants were allocated randomly into either the HI-group or the LI-group using a computer random number generator with randomly varied permuted blocks of 4 to 6 and an equal 1:1 allocation ratio, stratified by study site. Randomization took preferably place per 24 participants per site simultaneously, resulting in balanced HI-groups and LI-groups of 12 participants each and equal starting point of the intervention. To promote blinding and adherence, and inspired by U.S. POINTER [26], we deliberately avoid the label ‘control group’, but rather designate both groups as receiving some form of lifestyle intervention, differing however in structure and intensity. Personnel collecting the primary outcome measures (cognition) and researchers conducting the statistical analyses are blinded to group allocation.

### Intervention program

#### High-intensity intervention

The HI-group follows a personalized, supervised and hybrid intervention, addressing a combination of 7 lifestyle components and 1 nutritional product. The 2-year intervention consist of 24 monthly online group meetings (duration: 90-min; 2–4 lifestyle domains addressed each meeting) and 8 three-monthly group meetings at the study site (duration: 120 min; 4–5 lifestyle domains addressed each meeting), all guided by a lifestyle coach, resulting in a total of 32 group meetings. The group meetings are supplemented with 6 individual sessions with a lifestyle coach to personalize the intervention (phone/video consultations; duration: 10–60 min; one or more specific lifestyle domains addressed each meeting depending on the needs and goals of the participant following the principles of motivational interviewing). Finally, participants have access to a digital intervention platform to engage in individual online sessions (duration: 5 min up to four hours per week) and access to additional, carefully selected or custom-made training material (e.g. videos, apps) throughout the intervention period. To avoid overload and foster adherence, lifestyle components are initiated in a stepwise manner and the burden of the intervention is deliberately distributed over 24 months. See Table 1 and Additional file 1 for an overview of all intervention domains and activities. Each group is guided through the intervention by a local intervention team, consisting of an experienced lifestyle coach and a study navigator. The (certified) lifestyle coach is responsible for the actual execution of all intervention modules and activities as well as personalizing the intervention. Professional profiles differed, but the majority of lifestyle coaches was physiotherapist or dietitian. The study navigator (no specific background; e.g., study nurse or junior researcher) is the site-specific primary contact point for participants and provides (ad-hoc) individual support and ongoing encouragement to achieve maximum adherence and reduce dropouts.

**Table 1:**
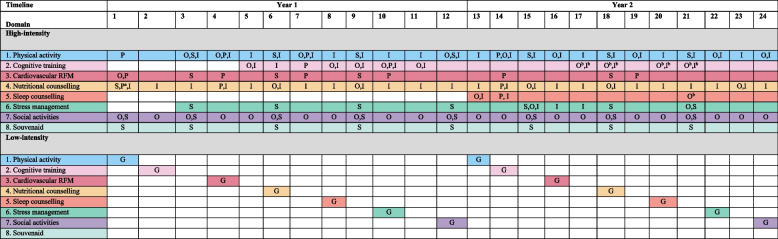
Overview of lifestyle domains in the FINGER-NL high-intensity and the low-intensity group

* Abbreviations*: *G* General, online health-related information, *I* Individual online session, *O* Online group meeting, *P* Personal lifestyle coach session, *RFM* Risk factor management, *S* Study-site group meeting

^a^Two sessions in one month

^b^Booster session

#### Low-intensity intervention

The LI-group receives general lifestyle-related health information covering all domains of the high-intensity intervention except the nutritional product. In the course of the study duration, 14 information leaflets are shared with participants, i.e., one in each intervention year for each of the seven lifestyle intervention domains (see Table 1). The information leaflets are provided via the digital intervention platform.

### Digital intervention platform

To improve trial efficiency (and in anticipation of possible (future) COVID-19 restrictions which were still highly probable at the time of study start), participants have access to a personal intervention environment via a digital platform (Ivido). This digital platform is used to facilitate the trial workflow and execution of the intervention. Participants in the HI-group have access to: (1) a personal landing page/dashboard including a general overview of participants’ personal health profile); (2) online aspects of the intervention (e.g. video instructions for home exercise, training material, videos, weekly information or motivational snippets to boost adherence) and receive personalized information and easy access to integrated applications which support the intervention; (3) chat function to communicate with the research team; (4) insight into intermediate outcomes (e.g. blood pressure, body mass index (BMI)). Furthermore, the platform is used for data-collection such as the administration of questionnaires/measurements and to measure adherence to the several intervention domains (e.g. number of tasks completed). For participants in the LI-group access to the online dashboard is limited to general lifestyle-related health information and administration of questionnaires/measurements.

To foster adherence, both groups receive regular newsletters via e-mail. At the two follow-up outcome measurements, participants receive small gift vouchers (€10-€20).

### Baseline and outcome measurements

All study participants undergo a baseline measurement and two follow-up outcome measurements at 12 months (follow-up 1) and 24 months (follow-up 2) after randomization at the research site. The baseline and follow-up outcome measurements include an assessment of demographics (e.g., age, gender, level of education, socioeconomic status; baseline only), medical history and medication (including items on smoking and alcohol consumption), cognitive testing, clinical measures, blood sampling (e.g., APOE e4 carriership) and questionnaires (See Table 2). At baseline only, we used the Montreal Cognitive Assessment (MOCA) to assess global cognitive performance [27]. All measurements/tests were administered by trained personnel. For personnel collecting the primary outcome measures (cognition) regular intervision meetings were set up.

**Table 2:** FINGER-NL data-collection

Month	0	12	24	Type of outcome
**Cognitive tests**				
Montreal Cognitive Assessment	x			Exploratory
15-Word Verbal Learning Test	x	x	X	Primary, secondary
Digit Symbol Substitution Test	x	x	X	Primary, secondary
Wechsler Adult Intelligence Scale digit span	x	x	X	Primary, secondary
Animal fluency	x	x	X	Primary, secondary
**Clinical measures**				
Height and weight	x	x	X	Secondary
Blood pressure	x	x	X	Secondary
Waist and hip circumference	x	x	X	Secondary
Medical history and medication^a^	x	x	X	Secondary
Maximum grip strength	x	x	X	Secondary
7 days Accelerometry (ActiGraph GT3X)^b^	x	x	x	Secondary
**Blood-based markers**				
Cholesterol (total, HDL, LDL + triglycerides)	x	x	x	Secondary
HbA1c	x	x	x	Secondary
Plasma glucose	x			Secondary
Creatinine	x			Secondary
APOE genotyping	x			Exploratory
Aβ 40/42, p-tau, NfL, GFAP, BDNF	x		x	Secondary
**Questionnaires/other**				
A-IADL-Q^c^	x	x	x	Secondary
LIBRA	x	x	x	Secondary
EQ-5D-5L	x	x	x	Secondary
MCLHB-DRR	x		x	Exploratory
Metamemory in Adulthood Questionnaire	x	x	x	Secondary
Hill-Bone Medication Adherence Scale	x	x	x	Secondary
MIND-NL Eetscore FFQ^d^	x	x	x	Secondary
3-day food diary via Traqq app	x	x	x	Secondary
SQUASH	x	x	x	Secondary
LASA Sedentary Behavior Questionnaire	x	x	x	Secondary
Pittsburgh Fatigability Scale	x	x	x	Secondary
SARC-F Sarcopenia Questionnaire	x	x	x	Secondary
7 days Physical Activity Record^b^	x	x	x	Secondary
Insomnia Severity Index	x	x	x	Secondary
7 days Sleep diary	x	x	x	Secondary
Pittsburg Sleep Quality Index	x			Secondary
Five Facet Mindfulness Questionnaire	x	x	x	Secondary
Perceived Stress Scale	x	x	x	Secondary
Lubben Social Network Scale	x	x	x	Secondary
De Jong Gierveld Loneliness Scale	x	x	x	Secondary
9-item Patient Health Questionnaire (PHQ-9)	x	x	x	Secondary
Cognitive Reserve Index Questionnaire (CRIq)	x	x	x	Secondary

*Abbreviations*: *Aβ 40/42* amyloid-beta 40/42, *A-IADL-Q* Amsterdam Instrumental Activity of Daily Living Questionnaire, *APOE* Apolipoprotein E, *BDNF* brain derived neurotrophic factor, *EQ-5D-5L* 5-level EuroQol-5D, *FFQ* Food Frequency Questionnaire, *GFAP* glial fibrillary acidic protein, *HbA1c* hemoglobin A1c, *HDL* high-density lipoprotein, *LDL* low-density lipoprotein, *LIBRA* LIfestyle for BRAin health, *MCLHB-DRR* Motivation to Change Lifestyle and Health Behavior for Dementia Risk Reduction scale, *MIND* Mediterranean-DASH Intervention for Neurodegenerative Delay, *NfL* neurofilament light, *p-tau* phosphorylated tau, *SQUASH* Short QUestionnaire to ASsess Health-enhancing physical activity

^a^Including items on smoking and alcohol consumption

^b^Only measured among subgroup of participants (study site Groningen; *n* = 240)

^c^Completed by study partner

^d^Monthly assessment

### Primary outcome

The primary outcome is the 2-year change from baseline in a global cognitive composite score derived from subtest scores from the neuropsychological test battery (NTB). The NTB includes (i) 15-Word Verbal Learning Test delayed recall (episodic memory) [28], (ii) Digit Symbol Substitution Test 90 seconds (processing speed) [29], (iii) Wechsler Adult Intelligence Scale digit span backwards [30], and (iv) semantic fluency (animals; both attention and executive functions) [31, 32]. The choice for this subset was based on consultations with neuropsychologists to cover the cognitive domains attention and executive functioning, processing speed and memory, their sensitivity to early cognitive changes in older individuals, administration time, and suitability for harmonization with other large multidomain intervention studies. The raw scores of individual tests will be transformed into standardized z-scores using the cohort-wide means and standard deviations (SD) at baseline (with higher scores suggesting better performance). The NTB total score is obtained by averaging the z-scores of the four individual tests and re-standardization. The minimum number of necessary individual tests was set to 3 out of 4 for calculating the NTB total score.

### Secondary outcomes

Secondary outcome measurements include 2-year change on the individual cognitive test performances (see above), instrumental activities of daily living (Amsterdam Instrumental Activity of Daily Living Questionnaire (A-IADL-Q) [33]), quality of life (5-level EuroQol-5D (EQ-5D-5L) [34]), modifiable dementia risk (‘LIfestyle for BRAin health’ (LIBRA) score [23, 24]), and several intervention-specific outcomes (physical activity (maximum grip strength, physical activity (SQUASH questionnaire [35]), sedentary behavior (LASA Sedentary Behavior Questionnaire [36]) and sarcopenia (SARC-F Sarcopenia Questionnaire [37])), fatigability (Pittsburgh Fatigability Scale [38]), 7 days Accelerometry (ActiGraph GT9X) combined with 7 days Physical Activity Record (Groningen study site only), cognitive training (cognitive function, meta-memory (Meta-memory in Adulthood Questionnaire [39])), cardiovascular risk factor management (blood pressure, cholesterol (total, HDL, LDL + triglycerides), blood glucose (HbA1c), waist circumference, BMI, medication adherence (Hill-Bone Medication Adherence Scale [40])), nutritional counselling (nutritional intake (Traqq app [41]), adherence to MIND-NL-Eetscore Food Frequency Questionnaire [42]), sleep counselling (sleep behavior (7-day sleep diary), insomnia (Insomnia Severity Index [43])), stress management (mindfulness (Five Facet Mindfulness Questionnaire [44]), perception of stress (Perceived Stress Scale [45])), and social activities (perceived social support (Lubben Social Network Scale [46]), emotional and social loneliness (De Jong Gierveld Loneliness Scale [47])). In addition, we will analyze blood-based biomarkers for Alzheimer’s disease (Aβ42/40, p-tau), axonal damage (NfL), astrocytes activity/injury or stress (GFAP) and brain plasticity (BDNF).

### Exploratory outcomes (heterogeneity of treatment effects)

As an exploratory analysis, we investigate whether the effectiveness of the intervention with respect to the primary and secondary cognitive outcomes is modified by demographics (age, gender, years of education, socioeconomic status), baseline LIBRA score, baseline cognitive performance (MOCA), baseline scores of the Motivation to Change Lifestyle and health Behaviours for Dementia Risk Reduction scale, APOE e4 carriership, presence of a first-degree relative with dementia, presence of subjective cognitive decline/memory complaints and blood-based biomarkers for Alzheimer’s disease.

### Sample size

Sample size calculation was based on the primary outcome (NTB total score). In the original FINGER trial [8], an effect size of 0.127 (Cohen’s d) was reported for the 2-year change from baseline on the NTB total score following a multidomain lifestyle intervention. In the LipiDiDiet trial [13], an effect size of 0.17 (Cohen’s d) was reported for the 2-year change from baseline on the NTB total score following an intervention with Souvenaid. As FINGER-NL combines a multidomain lifestyle intervention with Souvenaid, an effect size (Cohen’s d) of 0.20 (SD = 1) for NTB total score was anticipated for the 2-year change from baseline on the NTB total score in the HI-group compared to the LI-group. With a power of 90%, 2-sided significance level of α = 0.05 and an anticipated drop-out rate of 12.5%, a total of 1,206 subjects (603 per group after randomization) was considered adequate to detect the desired effect size.

### Statistical analyses

In alignment with other WW-FINGERS studies, we will test for change in the primary and secondary outcome variables, both within-group and between-group using linear mixed models with random effects for intercept (individuals) and slope (time). Time and randomization group will be entered as fixed effects, study site will be included as fixed covariate. Change in the outcome variables will be modelled by including the interaction term between randomization group and time as fixed effect. The intention-to-treat principle will be used. Missing primary and secondary outcome data will be handled through restricted maximum likelihood (REML) estimation of expected scores in the mixed models. Given the size of FINGER-NL, no additional covariates will be included in the primary model, and differences between individuals will be treated as random nuisance parameters in the REML model [48]. For the primary outcome, the level of significance will be set at 0.05 (two-sided). The Benjamini–Hochberg correction will be used to account for multiple comparisons of correlated secondary and exploratory outcomes.

## Results

Recruitment started in January 2022 and was completed in May 2023. 2,844 persons expressed their interest in participation and 2,057 underwent telephone screening. A sample of *N* = 1,210 participants were randomized, with equal distribution across study sites (Amsterdam = 248; Maastricht = 242; Wageningen = 240; Groningen = 240; Nijmegen = 240). Baseline measurements were completed in June 2023. The mean age of the randomized participants was 67.6 years (SD: 4.6), 64% were female, 61% high educated, and the mean MOCA score was 26.7 (SD: 2.1). In terms of non-modifiable risk factors, 66% of the participants indicated to have a first-degree family history of dementia and 74% reported subjective memory complaints. Regarding modifiable risk factors, several lifestyle and cardiometabolic risk factors were present at baseline such as physical inactivity (89%), low mental/cognitive activity (50%), low social engagement (39%), hypertension (39%) and high alcohol consumption (39%). The mean body mass index of participants was 28.3 (SD: 4.2) and the total serum cholesterol was 5.4 mmol/L (SD: 1.2). The mean LIBRA score of the sample was 1.3 (SD: 2.7). See Table 3 and Additional file 2 for an overview of the baseline characteristics. The last participant’s, last visit is expected in June 2025.

**Table 3:** Baseline characteristics of participants randomized to the trial (*N* = 1,210)

**Variable**	**Participants with information available**	
**Demographics**		
Age at baseline visit, mean (SD)	1210	67.6 (4.6)
Female, n (%)	1210	771 (63.7)
Educational level, n (%)^a^	1209	
Low		170 (14.1)
Medium		304 (25.1)
High		735 (60.8)
Married or cohabiting, n (%)	1210	827 (68.4)
**Global cognitive performance**		
MOCA, mean (SD)	1208	26.7 (2.1)
**Non-modifiable dementia risk factors**		
First-degree family history of dementia, n (%)	1209	794 (65.7)
Subjective memory complaints, n (%)	1209	893 (73.9)
**Modifiable dementia risk factors**		
Current smoking, n (%)	1210	52 (4.3)
High alcohol consumption, n (%)^b^	1136	447 (39.4)
Physical inactivity, n (%)^c^	1181	1053 (89.2)
Low mental/cognitive activity, n (%)^d^	1190	590 (49.6)
MIND diet adherence score, mean (SD)^e^	1136	8.4 (1.8)
Sleep, n (%)^f^	1187	
Absence of insomnia		671 (56.5)
Sub-threshold clinical insomnia		384 (32.4)
Clinical insomnia		132 (11.1)
Low social engagement, n (%)^g^	1187	468 (39.4)
Depression, n (%)^h^	1210	148 (12.2)
Systolic BP ≥ 140 mmHg / diastolic BP ≥ 90 mmHg, n (%)	1205	475 (39.4)
Total cholesterol, mmol/L, mean (SD)	1206	5.4 (1.2)
Body mass index, mean (SD)	1209	28.3 (4.2)
**Modifiable dementia risk score**		
LIBRA, mean (SD)^i^	1117	1.3 (2.7)

*Abbreviations*: *BP* blood pressure, *LIBRA* LIfestyle for BRAin health, *MIND* Mediterranean-DASH Intervention for Neurodegenerative Delay, *MOCA* Montreal Cognitive Assessment, *SD* standard deviation

^a^Education level was categorized into low, medium and high based on the International Standard Classification of Education (ISCED 2011) guidelines

^b^A score of < 10 on the alcohol consumption item of the MIND-NL Eetscore Food Frequency Questionnaire, indicating more than 7 glasses of alcohol per week

^c^Non-adherence to the World Health Organization guidelines on physical activity (at least 300 min of moderate aerobic activity per week) measured with the Short QUestionnaire to ASsess Health-enhancing physical activity (SQUASH)

^d^A score of < 130 on the Cognitive Reserve Index Questionnaire (CRIq)

^e^Measured with the MIND-NL Eetscore Food Frequency Questionnaire (theoretical range: 0–15; observed range: 2–14)

^f^Based on the Insomnia Severity Index with a score of ≤ 7 (absence of insomnia), 8–14 (sub-threshold clinical insomnia), ≥ 15 (clinical insomnia)

^g^A score of ≥ 3 on the De Jong Gierveld Loneliness Scale or a score of ≤ 14 on the Lubben Social Network Scale

^h^A score of ≥ 10 on the Patient Health Questionnaire (PHQ-9) and/or a self-reported diagnosis of depression as indicated on the medical history questionnaire

^i^LIBRA score theoretical range: − 5.9 to 12.7; observed range: − 4.9 to 10.2, with higher scores indicating higher dementia risk

## Discussion

We described the design and baseline characteristics of the study population of FINGER-NL, a new-generation multidomain lifestyle intervention on cognitive functioning in Dutch older adults at risk of cognitive decline. The study design is based on the original FINGER trial and optimized under local (Dutch, post-COVID-19) settings [8, 12]. Main adjustments are the hybrid design (online, on-site) and the addition of three more lifestyle domains (i.e. sleep counseling, stress management, and social activities) and a nutritional product to the intervention.

The preparatory phase of FINGER-NL took place during the COVID-19 pandemic. Using a survey, we found that the lockdown measures spurred both improvements and decline in people’s lifestyles. This provided us with the knowledge that there was rationale for FINGER-NL to start during COVID-19 times, and reason to believe that even during lockdown, improvement of lifestyle is feasible [49]. To be resilient to putative future lockdown measures, we designed FINGER-NL to have both online and on-site intervention components.

The digital platform is a central feature of the intervention. A previous mixed-methods study has shown that web-based lifestyle programs can positively influence brain health outcomes and have the potential to help maintain brain health [50]. In the Netherlands, internet accessibility among older adults is high [51]. Adding a digital platform to the intervention also offers new opportunities for trial design, and it is expected that this will meet the needs and wishes of future older generations even better, rendering such interventions more sustainable. On the other hand, difficulties with accessing and using the platform could have a negative effect on the intervention adherence. As a solution, during our first in-person meeting, the study personnel introduces the platform and provides hands-on training. In addition, instruction manuals and a helpdesk are available throughout the intervention. One side effect of the COVID-19 pandemic is that older individuals’ use of the internet and digital technology increased even further compared to pre-pandemic levels [52].

A known challenge of longer-term trials is the adherence of participants to the intervention. Inspired by the original FINGER trial and U.S. POINTER [8, 26], participants in the high-intensity intervention arm have been placed in groups of approximately 12 individuals to encourage social/peer support among participants. Another challenge was the recruitment of a large number of study participants necessary for this trial. We primarily recruited via the Dutch Brain Research Registry, which is an online registry of individuals interested in participating in neuroscience studies [22]. It currently includes over 40.000 registrants, mainly middle‐to‐late age cognitively normal elderly. A dedicated recruitment campaign was launched for current and new registrants. The registry was then used to prescreen participants based on demographic, lifestyle and (other) risk factors for dementia and to invite people for further telephone screening performed by one of the five FINGER-NL study sites. We made the choice to pre-screen based on self-reported risk factors of cognitive decline. Whilst this may have been less precise, it was effective in keeping recruitment feasible. Based on the baseline characteristics, this strategy indeed resulted in a population with sufficient potential for prevention. The room for improvement in terms of lifestyle (mean LIBRA score) is slightly higher in the FINGER-NL sample compared with the original FINGER trial [21].

## Conclusions

Results of FINGER-NL will provide further insight into the efficacy of a multidomain lifestyle intervention combined with a nutritional product to prevent cognitive decline in older adults. Findings can guide and inspire other countries to set up new-generation of combination therapies with lifestyle intervention and pharmacological treatments.

### Supplementary Information

Supplementary Material 1.

## Data Availability

Due to ethical restrictions and privacy regulations, FINGER-NL data is currently not publicly available. Interested researchers can send a data request to the FINGER-NL Data Access Committee. Upon reasonable request, the data analysis protocols can be made available from the corresponding author.

## Abbreviations

BMI: Body mass index
FINGER: Finnish Geriatric Intervention Study to Prevent Cognitive Impairment and Disability
HI: High-intensity
LI: Low intensity
LIBRA: LIfestyle for BRAin health
MIND: Mediterranean-DASH Intervention for Neurodegenerative Delay
MOCA: Montreal Cognitive Assessment
NTB: Neuropsychological test battery
SD: Standard deviation
TICSm: Modified Telephone Interview for Cognitive Status battery
