# Supplementary material for: A multidomain lifestyle intervention to maintain optimal cognitive functioning in Dutch older adults—study design and baseline characteristics of the FINGER-NL randomized controlled trial
Source: Alzheimers Res Ther. 2024 Jun 13;16:126. doi: 10.1186/s13195-024-01495-8 (PMC11170777; doi:10.1186/s13195-024-01495-8)
Supplement: Supplementary file 1 — Supplementary Material 1. [file 13195_2024_1495_MOESM1_ESM.docx]

**Additional file 1**. Outline of the FINGER-NL intervention domains.

*Physical activity*

The goal of the physical activity module is to meet the 2020 World Health Organization (WHO) Physical Activity guidelines [1]. These guidelines recommend engaging in physical activity of moderate intensity for at least 150 to 300 minutes or 75 to 150 minutes of vigorous intensity aerobic physical activity, or an equivalent combination of moderate- and vigorous-intensity activity, per week. Moreover, the guidelines recommend to perform muscle-strengthening activities at moderate or greater intensity that involve all major muscle groups at least twice a week combined with balance exercises, and limit the amount of sitting time. During the 2-year intervention period, physical activity is addressed in 9 online group meetings, 7 study-site meetings and 5 personal lifestyle coach sessions (see Table 1). During these group meetings, guided by the lifestyle coach, information about the importance of being physically active and limiting sedentary behavior will be provided. Furthermore, muscle-strengthening exercises combined with balance and stretch exercises will be explained and performed. In addition, participants are asked to perform weekly individual sessions of physical exercises and muscle-strengthening exercises (facilitated via the online platform). Participants can login on website providing videos of the lifestyle coach showing the light, moderate and vigorous muscle-strengthening exercises (see www.finger-beweeg.nl). Individual goals are set in dialogue with the lifestyle coach and depend on individual level of fitness, regular habits and personal preferences. Exercises for both aerobic physical activity and muscle strengthening gradually increase in frequency, intensity and duration over time. In addition, participants are encouraged to use the Ommetje-app.

*Cognitive training*

The cognitive training module consists of two components: strategy training and functional training. The strategy training is based on the “Keep your brain fit” psychoeducational program [2]. The goal of the strategy training is to provide information about changes in the aging brain, to teach strategies to better deal with cognitive changes that are part of normal aging, and to teach how to apply these strategies in daily life. The functional training aims to improve and maintain cognitive functions using Brain-HQ. The duration of the cognitive training module is 4 months and the module will be addressed in 5 online group meetings, 2 personal lifestyle coach sessions, supplemented with 6 individual sessions provided via the digital intervention platform (see Table 1). During the second intervention year, two-month booster sessions are provided on two occasions, each including two online group meetings and two individual sessions. The strategy training comprises two individual online modules: ‘memory’, and ‘working effectively’, with a duration of 2.5 hours each, to be completed at participant’s own pace within a two-month timeframe. Next to a general introduction about the strategy training, participants will set personal goals together with the lifestyle coach. The functional training is provided through BrainHQ and consists of three weekly individual online sessions of 30 minutes each. During each individual online session, an alternating combination of exercises is presented. These exercises are adaptive in difficulty to facilitate a maximum (individual) training effect and progress is monitored by the lifestyle coach or study guide.

*Cardiovascular risk factor management*

The goal of the cardiovascular risk factor management module is to achieve adherence to the Dutch guideline for cardiovascular risk management [3]. Cardiovascular risk factor management is addressed during 1 online group meeting, 4 at study-site group meetings and 6 personal lifestyle coach sessions. The intervention is based on regular monitoring of and informing about individual risk factors. Next to providing the participants with information on cardiovascular risk factors, clinical cardiovascular measurements (e.g. body mass index, waist-hip circumference ration, blood pressure) are also performed at 4 study-site group meetings. If indicated based on the clinical blood pressure measurement (systolic blood pressure between 140-179 mmHg), 1-week of home blood pressure measurements are performed. Participants are actively informed about results of each of the measurements via the online platform and during personal sessions with the lifestyle coach. No drugs are prescribed within the trial. General practitioners (GPs) of the participant are actively engaged in the trial if indicated. The GP receives a letter with the results of the measurements when changes in medication are indicated based on the Dutch guidelines for cardiovascular risk management. Additionally, the lifestyle coach will advise participants to see their GP if (change in the) treatment for high blood pressure or high cholesterol is indicated.

*Nutritional counselling*

The goal of nutritional counselling is to support participants to adhere to the Mediterranean-DASH Intervention for Neurodegenerative Delay (MIND)-NL diet [4] and the Dutch recommendation for vitamin D intake. During the 2-year intervention period, nutritional counselling is addressed in 5 online group meetings, 1 at study-site group meeting and 4 personal lifestyle coach sessions (see Table 1). Group sessions consist of an introduction to the MIND diet, information and support for facilitating changes in dietary behavior, peer discussions and practical exercises. During the personal sessions with the lifestyle coach, the diet of participants will be monitored and adjusted if needed based on their dietary history and monthly diet assessment (MIND-NL Eetscore Food Frequency Questionnaire) [5]. To comply with national vitamin D recommendations, participants are advised (and supported by means of an dedicated gift card) to take additional vitamin D: 10 μg/day for all women aged 60-69 years and for men with medium skin tone (non-Caucasian); 20 μg/day for men and women ≥70 years.

*Sleep counselling*

The goals of sleep counselling are to improve sleep quality and reduce insomnia if present within the participants. Sleep counselling is addressed in a two-month period and consist of 1 online group meeting, 1 personal lifestyle coach session, supplemented with individual sessions provided via the digital intervention platform (see Table 1). In addition, there is a booster session, which includes an online group meeting. Sleep counselling is based on a validated, guided, online Cognitive Behavioural Therapy for Insomnia (CBT-I), provided via i-Sleep ([www.i-sleep.nl](http://www.i-sleep.nl)) [6]. I-sleep consists of five online individual sessions, each consisting of theory (both text and video) and exercises (i.e. sleep diary and setting bedtimes). Participants are instructed to complete one session every week and are provided with online feedback from the lifestyle coach based on their individual goals, homework assignments and personal needs. As I-sleep was initially developed for people suffering from insomnia, an adjusted version was developed for the current study population by making small textual changes; the content, however, remained unaltered.

*Stress management*

The goal of this domain is to support participants in dealing with daily stress and making brain-healthy choices, by means of mindfulness exercises focusing on attention control, emotion regulation and self-awareness [7]. Stress management is addressed in two online group meetings, eight study-site group meetings and supplemented with individual online sessions provided via the digital intervention platform (see Table 1). A selection of exercises from the VGZ Mindfulness Coach (e.g. 3-minute breathing exercise, body scan) are included in the digital intervention platform [8]. The exercises consist of self-guided training with 6 individual online sessions per week of approximately 15 minutes, and a diary to reflect on their weekly moments of stress or relaxation. After the two months, participants were encouraged to continue the mindfulness exercises via the VGZ mindfulness coach app. In addition, the 3-minute breathing exercises are a regular component of the study-site group meetings.

*Social activities*

Previous multi-modal lifestyle interventions have suggested that the social component may be the most relevant component in achieving efficacy. Therefore, the goal of the social activity domain is to create a group feeling and to stimulate social interaction between participants thereby increasing study retention, intervention adherence and personal motivation. To ensure feasibility and foster social activities, participants will be placed in groups of approximately 12 individuals that progress through the intervention together. Social activity is promoted throughout the 2-year intervention period by means of a fixed structure of each group meeting (both online and on-site) including a team-building activity and/or a coffee-tea moment (social gathering), promoting social contacts within the group (creation of a buddy-system and a WhatsApp group), and facilitation of a (quarterly/monthly) walking club.

*Souvenaid*

Souvenaid® is a nutritional product that contains the nutrient combination Fortasyn Connect designed to address specific nutritional needs in early Alzheimer’s disease, including lower levels of circulating nutrients required for brain phospholipid synthesis [9] and an elevated resting energy expenditure [10]. Previous studies showed benefits on cognitive and behavioral outcomes and on brain shrinkage in individuals with mild cognitive impairment [11, 12]. It was shown to have additional effects to a multimodal FINGER lifestyle intervention in the MIND-AD mini trial. It is conceivable that daily consumption of Souvenaid could help maintain cognitive function in elderly at risk of cognitive decline as well. Participants are recommended to add one serving of Souvenaid (125 mL) to their daily diet during the entire intervention period. Participants can chose from two different flavors (strawberry, vanilla) to match personal preferences.

**References**

1. Bull FC, Al-Ansari SS, Biddle S, Borodulin K, Buman MP, Cardon G, Carty C, Chaput JP, Chastin S, Chou R, Dempsey PC, DiPietro L, Ekelund U, Firth J, Friedenreich CM, Garcia L, Gichu M, Jago R, Katzmarzyk PT, Lambert E, Leitzmann M, Milton K, Ortega FB, Ranasinghe C, Stamatakis E, Tiedemann A, Troiano RP, van der Ploeg HP, Wari V, Willumsen JF. World Health Organization 2020 guidelines on physical activity and sedentary behaviour. Br J Sports Med. 2020 Dec;54(24):1451-1462. doi: 10.1136/bjsports-2020-102955.
2. Reijnders JS, Geusgens CA, Ponds RW, van Boxtel MP. "Keep your brain fit!" Effectiveness of a psychoeducational intervention on cognitive functioning in healthy adults: A randomised controlled trial. Neuropsychol Rehabil. 2017 Jun;27(4):455-471. doi: 10.1080/09602011.2015.1090458.
3. Nederlands Huisartsen Genootschap. NHG-Standaard Cardiovasculair risicomanagement (M84). Versie 4.0, juni 2018: <https://richtlijnen.nhg.org/standaarden/cardiovasculair-risicomanagement>
4. van den Brink AC, Brouwer-Brolsma EM, Berendsen AAM, van de Rest O. The Mediterranean, Dietary Approaches to Stop Hypertension (DASH), and Mediterranean-DASH Intervention for Neurodegenerative Delay (MIND) Diets Are Associated with Less Cognitive Decline and a Lower Risk of Alzheimer's Disease-A Review. Adv Nutr. 2019 Nov 1;10(6):1040-1065. doi: 10.1093/advances/nmz054.
5. Looman M, Feskens EJ, de Rijk M, Meijboom S, Biesbroek S, Temme EH, de Vries J, Geelen A. Development and evaluation of the Dutch Healthy Diet index 2015. Public Health Nutr. 2017 Sep;20(13):2289-2299. doi: 10.1017/S136898001700091X.
6. Van der Zweerde T, Lancee J, Slottje P, Bosmans JE, Van Someren EJW, van Straten A. Nurse-Guided Internet-Delivered Cognitive Behavioral Therapy for Insomnia in General Practice: Results from a Pragmatic Randomized Clinical Trial. Psychother Psychosom. 2020;89(3):174-184. doi: 10.1159/000505600.
7. Tang YY, Hölzel BK, Posner MI. The neuroscience of mindfulness meditation. Nat Rev Neurosci. 2015 Apr;16(4):213-25. doi: 10.1038/nrn3916.
8. Russell-Williams J, Jaroudi W, Perich T, Hoscheidt S, El Haj M, Moustafa AA. Mindfulness and meditation: treating cognitive impairment and reducing stress in dementia. Rev Neurosci. 2018 Sep 25;29(7):791-804. doi: 10.1515/revneuro-2017-0066.
9. de Wilde MC, Vellas B, Girault E, Yavuz AC, Sijben JW. Lower brain and blood nutrient status in Alzheimer's disease: Results from meta-analyses. Alzheimers Dement (N Y). 2017 Jun 24;3(3):416-431. doi: 10.1016/j.trci.2017.06.002.111.
10. Doorduijn AS, de van der Schueren MAE, van de Rest O, de Leeuw FA, Hendriksen HMA, Teunissen CE, Scheltens P, van der Flier WM, Visser M. Energy intake and expenditure in patients with Alzheimer's disease and mild cognitive impairment: the NUDAD project. Alzheimers Res Ther. 2020 Sep 26;12(1):116. doi: 10.1186/s13195-020-00687-2.
11. Soininen H, Solomon A, Visser PJ, Hendrix SB, Blennow K, Kivipelto M, Hartmann T; LipiDiDiet clinical study group. 24-month intervention with a specific multinutrient in people with prodromal Alzheimer's disease (LipiDiDiet): a randomised, double-blind, controlled trial. Lancet Neurol. 2017 Dec;16(12):965-975. doi: 10.1016/S1474-4422(17)30332-0.
12. Soininen H, Solomon A, Visser PJ, Hendrix SB, Blennow K, Kivipelto M, Hartmann T; LipiDiDiet clinical study group. 36-month LipiDiDiet multinutrient clinical trial in prodromal Alzheimer's disease. Alzheimers Dement. 2021 Jan;17(1):29-40. doi: 10.1002/alz.12172.

**Additional file 2.** Additional baseline characteristics of participants randomized to the trial (N = 1,210)

|  |  |  |
| --- | --- | --- |
| **Variable** | **Participants with information available** |  |
| **Cardiometabolic risk factors** |  |  |
| Systolic BP, mmHg, mean (SD) | 1205 | 135.5 (15.7) |
| Diastolic BP, mmHg, mean (SD) | 1205 | 79.9 (9.5) |
| LDL-cholesterol, mmol/L, mean (SD) | 1204 | 3.2 (1.1) |
| HBA1c, mmol/mol, mean (SD) | 1200 | 39.7 (6.7) |
| eGFR, mean (SD) | 1204 | 84.8 (13.7) |
| **Self-reported medical history/medication use** |  |  |
| Coronary heart disease, n (%) | 1210 | 159 (13.1) |
| Diabetes, n (%) | 1210 | 105 (8.7) |
| Chronic kidney disease, n (%) | 1210 | 38 (3.1) |
| Antihypertensive drug(s), n (%) | 1210 | 679 (56.1) |
| Cholesterol-lowering drug(s), n (%) | 1210 | 481 (39.8) |
| **Cognition**^¶^ |  |  |
| 15-Word Verbal Learning Test delayed recall, mean (SD) | 1209 | 7.7 (3.5) |
| Digit Symbol Substitution Test 90 seconds, mean (SD) | 1205 | 50.3 (9.9) |
| Wechsler Adult Intelligence Scale digit span backwards, mean (SD) | 1209 | 6.5 (1.9) |
| Semantic fluency, mean (SD) | 1209 | 25.1 (5.6) |
| NTB total score, mean (SD) | 1209 | -0.0 (1.0) |

Abbreviations: BP = blood pressure; HbA1c = hemoglobin A1c; eGFR = estimated glomerular filtration rate; LDL = low-density lipoprotein; NTB = neuropsychological test battery; SD = standard deviation;
